# The Psychological Impacts of a COVID-19 Outbreak on College Students in China: A Longitudinal Study

**DOI:** 10.3390/ijerph17113933

**Published:** 2020-06-02

**Authors:** Hong Yan Li, Hui Cao, Doris Y. P. Leung, Yim Wah Mak

**Affiliations:** 1 Institute of Higher Education, Agricultural University of Hebei, Baoding 071001, China; lihongyan@hebau.edu.cn; 2 Capital Institute for Basic Education, Beijing Institute of Education, Beijing 100120, China; caohui@bjie.ac.cn; 3 School of Nursing, the Hong Kong Polytechnic University, Hong Kong, China; doris.yp.leung@polyu.edu.hk

**Keywords:** COVID-19, depression, anxiety, mood, college students, longitudinal study

## Abstract

An outbreak in Wuhan, China in late 2019 of a highly infectious new coronary pneumonia (COVID-19) led to the imposition of countrywide confinement measures from January to March 2020. This is a longitudinal study on changes in the mental health status of a college population before and after their COVID-19 confinement for the first two weeks, focusing on states of psychological distress, depression, anxiety and affectivity. The influence of possible stressors on their mental health were investigated, including inadequate supplies and fears of infection. Five hundred and fifty-five undergraduate students were recruited from Hebei Agricultural University in Baoding, China. The participants completed two online surveys—on anxiety and depression, and on positive and negative affect. One survey was conducted before the confinement and the other was conducted 15–17 days after the start of the confinement. Increases in negative affect and symptoms of anxiety and depression (*p*-values < 0.001) were observed after 2 weeks of confinement. Inadequate supplies of hand sanitizers, a higher year of study, and higher scores on anxiety and depression were common predictors of increased negative affect, anxiety, and depression across the confinement period. The results suggest that healthcare policymakers should carefully consider the appropriate confinement duration, and ensure adequate supplies of basic infection-control materials.

## 1. Introduction

New coronary pneumonia (COVID-19) has become a global public health emergency. By late December 2019, it became clear that a novel coronavirus was spreading through Wuhan, the capital of Hubei province in China [1]. A group of patients who had been exposed to the virus exhibited symptoms of fever, dyspnea, and severe pneumonia [2]. By 23 January 2020, 835 laboratory-confirmed cases of COVID-19 infection and 26 deaths were recorded by the Chinese Center for Disease Control and Prevention (China CDC) [3]. Those with the disease can have nonspecific symptoms, such as a dry cough, fever, muscle aches; or exhibit anything from no symptoms to severe pneumonia [4]. Confirmed cases have since been reported in more than 150 countries, with an estimated 7000 deaths around the world in March 2020 [5].

Numbers of diagnosed cases and deaths from COVID-19 were higher in Wuhan than in other cities in the early stage of the outbreak; and confinement, with travel restrictions, was first implemented in that city [6]. Other provinces throughout China successively followed suit during the month of January 2020. The centralized isolation of all suspected cases of COVID-19 infection was conducted in February 2020, and the opening of all schools in Wuhan for the new semester was postponed until further notice to control the epidemic [7]. 

By the end of February 2020, 79,824 confirmed cases of COVID-19 (66,907 in Hubei province, including 49,122 in Wuhan) and 2870 deaths (2761 in Hubei province, including 2195 in Wuhan) in 31 provinces and province-level regions of mainland China were reported to the National Health Commission [8]. After various infection-control measures were carried out, the epidemic in Wuhan began to ease by the middle of March, when only four new confirmed COVID-19 cases, one new case of suspected infection, and ten deaths were reported in Hubei province (all in Wuhan) [9].

Isolation and quarantine refer to the separating of people who have been diagnosed and suspected of having an infectious disease, respectively [10]. Both measures have been used to intercept the chain of infection and reduce the risk of infectious diseases breaking out in the community. Since the disease is highly contagious, almost all countries around the world have been significantly affected and have introduced confinement measures, including ordering citizens (even those with no symptoms of infection) to self-isolate at home, and closing businesses and schools [8]. The measures have been effective at preventing the transmission of COVID-19; however, concerns have arisen about their potential psychological impact [11]. Previous studies have found that people in quarantine reported higher rates of psychological symptoms, such as stress symptoms, depression, anxiety, and insomnia, compared to people not in quarantine or in the general population. The negative psychological effects were due to relatively long periods of quarantine, fears of infection, frustration, boredom, inadequate supplies, inadequate information, and stigma [11].

Most previous studies have focused on the psychological effects of people who were infected with COVID-19 [10], but a cross-sectional survey was conducted on the general populations of non-COVID-19 cases. The study was conducted in 194 cities in China and found that more than 50% of the 1210 respondents from the general population reported moderate to severe levels of psychological difficulties due to the COVID-19 outbreak, with about 16% suffering from symptoms of depression, 29% from anxiety, and 8% from stress [12]. The study also found that students were one of the groups experiencing a higher degree of psychological difficulty. Furthermore, only one previous study focused on the impact of COVID-19 on adolescents [13]. To the best of our knowledge, there has been no previous longitudinal study on the impact of COVID-19 during the confinement period on this population. 

Adolescence is a vital stage in the development and maintenance of mental wellbeing towards an independent adulthood. Emotional disorders such as anxiety, depression, and affectivity, can profoundly affect the health and school performance of adolescents [14]. We had been collecting data from a survey on the mental health of college students for a longitudinal study that began before the recognized outbreak of COVID-19. Taking advantage of this data, we surveyed the same population again during the confinement period. This study provides a longitudinal examination of the mental status of a population of college students in terms of negative and positive affects, as well as symptoms of anxiety and depression before and after 2 weeks of self-isolation at home. Additionally, investigated are the roles played by possible predictors of mental health status, including inadequate supplies of infection-control materials and the fear of infection.

## 2. Materials and Methods

The original longitudinal study included four waves of the survey, the aim of which was to observe the sleep habits and mental health status (affects, anxiety, and depression) of college students for two years. The first wave of the survey was conducted on 20 December 2019, which was before the outbreak of COVID-19. The plan was to conduct the second and third waves of the survey on the 4th to 6th days of February and on April 2020, to examine changes in the mental health of the college students over one semester. The fourth wave will be conducted one year after the completion of the third wave of the survey. To address the objective of the present study, that of determining the psychological impacts on the students of their confinement due to the COVID-19 outbreak, measures relating to supplies of infection-control materials and the fear of infection were added to the original survey. Each student was assigned a unique identifier when he/she completed the first wave of the survey. They were followed up in subsequent waves of the survey using this unique identifier. Data from the first and second waves of the survey were extracted for this study. Six hundred and twenty-four undergraduate students attending Hebei Agricultural University in Baoding, China, who had completed the first wave of the survey in December 2019 and had been affected by the confinement due to the COVID-19 outbreak, were recontacted to take part in the second survey. Their confinement began on 29 January 2020, at a time when all students were on their Chinese New Year break. The college students were required to stay at home under confinement, learn via on-line platforms, and send daily reports on their health and body temperature to officers of the school. The first survey was conducted on 20 December 2019 (BC: before confinement) and the second was conducted in February 2020, 15–17 days after the start of their home confinement (2WAQ: 2 weeks after the commencement of their confinement). A total of 555 (88.9%) students were ultimately included in the analysis because 69 did not provide the required information on some of the items, and were therefore excluded. The mean age of the participants was 19.6 (SD = 3.4), and most were female (*n* = 426, 76.8%) and in year one or two of their studies (99.1%). The study was approved by the Ethics Committee of Hebei Agricultural University (Ref: 20190309). Consent from each student was obtained at the beginning of each of the online surveys.

The immediate psychological consequences of the confinement were examined with reliable and valid self-reported questionnaires, which included the 10-item Positive and Negative Affect Schedule (PANAS) [15] and the 4-item Patient Health Questionnaire (PHQ-4) [16]. The PANAS contains two scales measuring mood: positive affect (PANAS-PA) and negative affect (PANAS-NA). Each mood scale has five items, with a Likert rating scale of 1 to 5. The PHQ-4 is an ultra-brief tool to measure symptoms of anxiety and depression; it has four items with a Likert rating scale of 0 to 3. Furthermore, eight items measuring inadequate supplies and fear of infection were included in the questionnaire used for 2 weeks after confinement (Appendix A). An exploratory factor analysis that was conducted on these eight items led to a 3-factor solution: fear of infection—general (three items), fear of infection—own risk (two items), and inadequate supplies of infection—control materials (three items). The Cronbach’s alpha of the three factors were 0.709, 0.928, and 0.564, respectively; thus, the three items in the last factor—inadequate supplies of masks, hand sanitizers, and access to groceries—were treated as individual variables in the analysis.

Statistical analyses were conducted using SPSS 25.0. Paired-*t* tests were used to examine the changes in PANAS-PA, PANAS-NA, and PHQ-4. Separate linear regression analyses were conducted to explore the factors associated with the PANAS-PA, PANAS-NA, and PHQ-4 mean scores at 2 weeks after confinement. The independent variables included in the regression models were fear of infection—general, fear of infection—own risk, and inadequate supplies of masks, hand sanitizers, and access to groceries, controlled for age, gender, year of study, and the score of the dependent variable before confinement.

## 3. Results

The participants had low mean scores for anxiety and depression (PHQ-4) and negative affect (PANAS-NA), and a moderate mean score for positive affect (PANAS-PA). Significant decrements were observed for negative affect (PANAS-NA) and symptoms of anxiety and depression PHQ-4 (Ps < 0.001), and a nonsignificant change on positive affect PANAS-PA (*p* = 0.107) after 2 weeks of confinement (Table 1). Table 2 shows the correlations among the studied variables, and it can be seen that the magnitudes of the correlations ranged from 0.006 to 0.680. 

The results of the three regression analyses for mean scores on positive affect (PANAS-PA), negative affect (PANAS-NA), as well as anxiety and depression (PHQ-4) after confinement are shown in Table 3, Table 4 and Table 5, respectively. The results revealed that in comparison to the scores obtained before the confinement, fear of infection—own risk had a significant association with reduced positive affect (PANAS-PA) in a negative sense (coefficient = −0.047, 95%CI = −0.080, −0.014); inadequate supplies of hand sanitizers was associated with increased negative affect (PANAS-NA) (coefficient = 0.043, 95%CI = 0.008, 0.078); and fear of infection—general (coefficient = 0.806, 95%CI = 0.008, 0.043) and inadequate supplies of alcohol hand rubs (coefficient = 0.031, 95%CI = 0.002, 0.060) were associated with an increase in anxiety and depression (PHQ-4) after 2 weeks of confinement, controlled for age, gender, and year of study, and the scores before confinement. There was no indication of multicollinearity, with all the VIFs ranging from 1.037 to 1.345 for the predictors in all three regression analyses.

## 4. Discussion

Several previous studies on patients with severe acute respiratory syndrome (SARS) and healthcare workers in hospitals had reported that quarantined persons harbored feelings of fear about the effects of their quarantine and about passing the disease on to loved ones [17,18,19,20,21,22]. Besides psychological problems, Hawryluck et al. [23] reported that some quarantined persons with exposure to SARS experienced health, emotional, and financial problems. However, Wang et al. [24] concluded that for college students, possibility of being exposed to H1N1 flu confinement did not have negative psychological effects.

Although the participants in this study were not themselves infected with COVID-19, they had potentially been exposed to this contagious disease. They followed enforced infection-control measures and needed to undergo confinement within their city and to stay at home in order to prevent the disease from being transmitted to the community. When undergoing confinement, they may have felt deprived of their liberty, experienced uncertainty over the status of their disease, and felt bored with being on their own, which may be associated with negative psychological impacts. If the duration of their confinement had been longer, the impact on their mental health might have been more severe.

In this survey, a range of factors associated with mean scores for psychological impacts was captured before and after 2 weeks of confinement from a cluster of college students. We also found that fear of infection—own risk increased by one score and that the PANAS-PA mean score significantly decreased by 0.05. In addition, fear of infection—general significantly increased the PHQ-4 by 0.806.

Inadequate supplies of hand sanitizers significantly increased both the PANAS-NA mean score by 0.043 and the PHQ-4 by 0.031. The study by Maunder et al. [17] suggested that the provision of adequate infection-control supplies to quarantined persons was a form of psychological support. Lee et al. [25] discovered in their study that healthcare workers experienced burnout, traumatic stress, anxiety, and depressive symptoms, even after the outbreak. However, Wu et al. [21] concluded that there was no association between quarantine and negative psychological effects. Furthermore, each year of study also significantly increased the mean score of the PANAS-NA by 0.123 and the mean score of the PHQ-4 by 0.136. This means that year-two college students would have a higher negative affect and higher levels of anxiety and depression than year-one students, most likely because the year-two curriculum is more tightly packed than the year-one curriculum; thus, the year-two students would be afraid that the confinement and having to learn online only would hinder their progress in their studies.

## 5. Strengths and Limitations of the Study

The experiences of confined college students were studied in this survey in order to understand their needs and concerns, and to explore the factors associated with the psychological impacts of their confinement. One strength of this survey is that the mental health survey data of the same cluster of college students were compared before the outbreak of COVID-19 and after 2 weeks of confinement to ensure temporal precedence of the findings of the study, meaning that background differences between the two comparison groups could be excluded. Moreover, we particularly studied the factor “fear of infection” in detail, by separating the factor into two items: fear of infection—general and fear of infection—own risk, as these were shown to have different impacts on the mental health status of the current sample of college students.

There are also several limitations to this survey. Our study group was comprised of undergraduate students, most of them female (about 77%); thus, the results might not be generalizable to the general population, including people working full-time who might be the main breadwinners of their families, or healthcare workers taking care of affected patients [26]. The quarantined participants of this survey were staying at home with their families in an extension of the Chinese New Year holiday. Therefore, they were not deprived of the company of their families and were still in a holiday mood; thus, the negative psychological impacts of being in quarantine might have weighed less heavily on them than on the general public or on healthcare providers. Nevertheless, the two waves of measures that have been taken include the total period of confinement, so the data set forth in this report should be reliable.

## 6. Conclusions

This study involving two waves of surveys explored the psychological impacts on college students in China of a massive confinement effort to prevent the transmission of COVID-19. The findings provide public health policy makers and mental health professionals with valuable information on the psychological impacts of an outbreak of contagious disease, which may assist them in making preparations for possible future outbreaks of novel contagious diseases. Although imposing confinement is an effective measure to control the wide spread of an infectious disease, our results suggest that it also leads to fear, anxiety, and depression, and has negative psychological impacts on the affected persons. The government should take measures to ensure that the experience of confinement is as tolerable as possible for people by determining a duration that is appropriate and ensuring that adequate amounts of basic supplies are available (such as food, water, and medical and hygiene supplies). Further studies can be conducted using a qualitative approach to explore factors associated with the psychological impacts on college students. A longitudinal study to examine the psychological impacts on students over the full confinement period is also warranted.

## Figures and Tables

**Table 1:** Changes in positive affect (PA) and negative affect (NA) in the Positive and Negative Affect Schedule (PANAS), and anxiety and depression in the 4-item Patient Health Questionnaire (PHQ-4) before and after confinement (*n* = 555).

	Before Confinement		After 2 Weeks of Confinement		After–Before Confinement		
	Range	Mean (SD)	Range	Mean (SD)	Range	Mean Difference (95% CI)	*p*-Value
**Positive Affect (PANAS-PA)**	1–5	3.21 (0.79)	1–5	3.26 (0.79)	−3.00–2.33	0.06 (−0.01, 0.12)	0.107
**Negative Affect (PANAS-NA)**	1–5	2.38 (0.79)	1–5	2.24 (0.80)	−4.00–4.00	−0.15 (−0.21, −0.08)	<0.001 *
**Anxiety and Depression (PHQ-4)**	1–4	0.95 (0.65)	1–4	0.76 (0.61)	−3.00–2.00	−0.19 (−0.24, −0.13)	<0.001 *

* *p* < 0.001.

**Table 2:** Correlations among the studied variables (*n* = 555).

	Possible Score Range	V1	V2	V3	V4	V5	V6	V7	V8	V9	V10	V11	V12	V13
**Positive Affect (Before confinement) (V1)**	1–5	1												
**Negative Affect (Before confinement) (V2)**	1–5	−0.102 *	1											
**Anxiety and Depression (Before confinement) (V3)**	1–4	−0.213 **	0.710 **	1										
**Positive Affect (After confinement) (V4)**	1–5	0.484 **	−0.224 **	−0.277 **	1									
**Negative Affect (After confinement) (V5)**	1–5	−0.195 **	0.567 **	0.414 **	−0.249 **	1								
**Anxiety and Depression (After confinement) (V6)**	1–4	−0.228 **	0.495 **	0.434 **	−0.310 **	0.680 **	1							
**Fear of Infection—general (V7)**	3–21	−0.077	0.089 *	0.047	−0.100 *	0.069	0.142 **	1						
**Fear of Infection—own risk (V8)**	2–20	−0.156 **	0.108 *	0.139 **	−0.188 **	0.107 *	0.077	0.160 **	1					
**Inadequate supplies of masks (V9)**	1–6	0.056	0.021	−0.013	−0.047	0.023	0.034	−0.004	0.005	1				
**Inadequate supplies of hand sanitizers (V10)**	1–7	−0.127 **	0.049	0.060	−0.113 **	0.107 *	0.113 **	−0.016	0.045	0.448 **	1			
**Inadequate access to groceries (V11)**	1–4	−0.030	0.095 *	0.112 **	−0.037	0.027	0.051	0.061	0.057	0.215 **	0.275 **	1		
**Age (V12)**	NA	−0.065	0.097 *	0.101 *	−0.026	0.126 **	0.127 **	0.020	−0.003	0.027	0.006	0.013	1	
**Gender (V13)**	1–2	0.007	−0.046	−0.027	0.033	−0.004	−0.041	−0.111 **	−0.089 *	0.126 **	0.066	0.042	−0.039	1
**Year of study (V14)**	1–4	−0.081	0.082	0.112 **	−0.071	0.128 **	0.180 **	0.155 **	0.054	−0.003	0.019	0.051	0.327 **	−0.052

* *p* < 0.001, ** *p* < 0.05.

**Table 3:** Linear regression model of positive affect (PANAS-PA) (*n* = 555).

Factor	Beta ^♐^	B ^#^ (95% CI ^‡^)	*p*-Value
Fear of infection—general	0.013	−0.012 (−0.034, 0.011)	0.297
Fear of infection—own risk	−0.105	−0.047 (−0.080, -0.014)	0.006 **
Inadequate supplies of masks	−0.064	−0.035 (−0.080, 0.010)	0.127
Inadequate supplies of hand sanitizers	−0.028	−0.012 (−0.050, 0.025)	0.512
Inadequate access to groceries	0.011	0.011 (-0.065, 0.086)	0.783
Age	−0.100	−0.023 (−0.042, −0.005)	0.013 **
Gender (ref = male)	0.027	0.050 (−0.088, 0.189)	0.475
Year of study	−0.013	−0.020 (−0.141, 0.100)	0.739
Score before quarantine	0.454	0.456 (0.381, 0.531)	<0.001 *
Adjusted R^2^ = 0.244; F(9544) = 20.828; *p*-value < 0.001			

* *p* < 0.001, ** *p* < 0.05, **^♐^** Beta = standardized coefficient; ^#^ B = coefficient; ^‡^ CI = confidence interval.

**Table 4:** Linear regression model of negative affect (PANAS-NA) (*n* = 555).

Factor	Beta ^♐^	B ^#^ (95% CI^‡^)	*p*-Value
Fear of infection—general	0.013	0.004 (−0.018, 0.025)	0.718
Fear of infection—own risk	0.044	0.020 (−0.012, 0.051)	0.222
Inadequate supplies of masks	−0.025	−0.014 (−0.056, 0.029)	0.531
Inadequate supplies of hand sanitizers	0.097	0.043 (0.008, 0.078)	0.016 **
Inadequate access to groceries	−0.051	−0.051 (−0.123, 0.021)	0.167
Age	−0.073	−0.017 (−0.035, 0.000)	0.055
Gender (ref = male)	0.034	0.065 (-0.068, 0.197)	0.339
Year of study	0.080	0.123 (0.008, 0.238)	0.036 **
Score before quarantine	0.556	0.557 (0.486, 0.627)	<0.001 *
Adjusted R^2^ = 0.332; F(9544) = 31.545; *p*-value < 0.001			

* *p* < 0.001, ** *p* < 0.05, ^♐^ Beta = standardized coefficient; ^#^ B = coefficient; ^‡^ CI = confidence interval.

**Table 5:** Linear regression model of anxiety and depression (PHQ-4) (*n* = 555).

Factor	Beta ^♐^	B ^#^ (95% CI ^‡^)	*p*-Value
Fear of infection—general	0.111	0.806 (0.008, 0.043)	0.005 **
Fear of infection—own risk	−0.007	−0.003 (−0.029, 0.0242)	0.851
Inadequate supplies of masks	0.007	0.003 (−0.032, 0.038)	0.873
Inadequate supplies of hand sanitizers	0.092	0.031 (0.002, 0.060)	0.035 **
Inadequate access to groceries	−0.030	−0.023 (−0.082, 0.037)	0.459
Age	−0.056	−0.010 (−0.025, 0.004)	0.173
Gender (ref = male)	−0.014	−0.020 (−0.130, 0.089)	0.715
Year of study	0.116	0.136 (0.041, 0.232)	0.005 **
Score before quarantine	0.414	0.389 (0.317, 0.461)	<0.001 *
Adjusted R^2^ = 0.215; F(9544) = 17.810, *p*-value < 0.001			

* *p* < 0.001, ** *p* < 0.05, ^♐^ Beta = standardized coefficient; ^#^ B = coefficient; ^‡^ CI = confidence interval.

## Appendix A

List of the eight items measuring inadequate supplies and fear of infection included in the 2WAQ questionnaire.

Fear of infection—

General Item 1: Please estimate the number of people will be infected by Covid-19:

Options for selection:

1 = “Less than 50,000”,

2 = “50,000–100,000”,

3 = “100,000–150,000”,

4 = “150,000–200,000”,

5 = “200,000–300,000”,

6 = “300,000–500,000”,

7 = “500,000–800,000”,

8 = “More than 800,000”.

Item 2: Please estimate when the transmission of Covid-19 will end:

Options for selection:

1 = “March”,

2 = “April”,

3 = “May”, 4 = “June”, 5 = “July”,

7 = “August”, 8 = “September or later”.

Item 3: Please estimate the number of death caused by Covid-19:

Options for selection:

1 = “Less than 1000”, 2 = “1000–1500”, 3 = “1500–2000”, 4 = “2000–3000”, 5 = “More than 3000”.

Fear of infection—

Own risk Item 4: Please estimate the possibility that you would be infected by Covid-19:

Options for selection:

1 = “0–10%”,

2 = “10%–20%”,

3 = “20%–30%”,

4 = “30%–40%”,

5 = “40%–50%”,

6 = “50%–60%”,

7 = “60%–70%”,

8 = “70%–80%”,

9 = “80%–90%”,

10 = “90%–100%”

Item 5: Please estimate the possibility that your family members would be infected by Covid-19: Options for selection:

1 = “0–10%”,

2 = “10%–20%”,

3 = “20%–30%”,

4 = “30%–40%”,

5 = “40%–50%”,

6 = “50%–60%”,

7 = “60%–70%”,

8 = “70%–80%”,

9 = “80%–90%”,

10 = “90%–100%”

Inadequate supplies of infection-control materials Item 6: Number of face masks do you have in your family (per person):

Options for selection:

1 = “0”,

2 = “1–2”,

3 = “3–5”,

4 = “6–10”,

5 = “11–20”,

6 = “More than 20”.

Item 7: Stock of bleach or 75% alcohol in your family could sustained:

Options for selection:

1 = ‘Used up’,

2 = ‘Less than 2 days’,

3 = “3 to 5 days”,

4 = “5 to 10 days”,

5 = “11 to 20 days”,

6 = “20 to 50 days”,

7 = ‘More than 50 days’

Item 8: Can you purchase groceries conveniently?

Options for selection:

1 = “Very convenient”,

2 = “Normal”

3 = “Somehow difficult”

4 = “Very difficult”.
